# Acute effects of a single recess physical activity session on sustained attention among junior high school students: a randomized controlled trial based on in-class task performance

**DOI:** 10.3389/fpsyg.2026.1781997

**Published:** 2026-05-18

**Authors:** Ximing Xiao

**Affiliations:** 1 School of Marxism, Wuhan University of Science and Technology, Wuhan, Hubei, China; 2 Public Opinion Research Center, Key Research Base of Humanities and Social Sciences in Hubei Universities, Wuhan, Hubei, China

**Keywords:** academic engagement, chain mediation, executive function, junior high school students, randomized controlled trial, recess physical activity, sustained attention

## Abstract

**Objectives:**

This randomized controlled trial explored the acute effects of a single recess physical activity session on junior high school students’ sustained attention, examined the chain mediating roles of executive function and academic engagement, and aimed to inform optimal recess arrangements.

**Methods:**

A total of 168 junior high school students from two public schools, stratified by grade and sex, were randomly assigned to an experimental group (20 min of moderate-to-vigorous physical activity during recess) or a control group (sedentary recess). Sustained attention was assessed at baseline and post-intervention using the d2 Test. Post-intervention measures included executive functioning (BRIEF-SR) and academic engagement (behavioral observations). Data were analyzed using a mixed-design ANOVA to assess the time and condition effects. Chain mediation was examined using the PROCESS macro (Model 6, 10,000 bootstrap samples) and structural equation modeling with maximum likelihood estimation.

**Results:**

Demographic analyses indicated higher physical activity intensity in males and better executive functioning in ninth graders, with no group differences in academic engagement or sustained attention at baseline. Mixed-design ANOVA revealed significant group × time interactions for sustained attention [F(1,166) = 18.42, *p* < 0.001, η^2^p = 0.10], executive functioning [F(1,166) = 12.56, *p* < 0.001, η^2^p = 0.07], and academic engagement [F(1,166) = 14.89, *p* < 0.001, η^2^p = 0.08]. The experimental group showed greater improvements in sustained attention (ΔM = 8.73 vs. −2.15), executive functioning (ΔM = 5.24 vs. 0.87, M_post = 54.3, SD = 8.1 vs. M_post = 49.1, SD = 7.9), and academic engagement (ΔM = 12.6% vs. 1.8%, M_post = 84.9%, SD = 10.3% vs. M_post = 73.2%, SD = 11.5%) than did the control group. Chain mediation modeling supported indirect effects through executive functioning alone (β = 0.09), academic engagement alone (β = 0.11), and their sequential pathway (β = 0.08, 95% CI [0.045, 0.128]), with total indirect effects explaining 60.9% of the total effect.

**Conclusion:**

A single moderate-to-vigorous physical activity session during recess acutely enhances junior high school students’ sustained attention, mediated sequentially by executive functioning and academic engagement. However, the simultaneous measurement of mediators suggests that these findings should be replicated using longitudinal designs featuring temporally separated assessments.

## 1 Introduction

With the deepening of quality-oriented education reform, the balanced development of junior high school students’ physical health and cognitive ability has become a key focus of educational practice. As an indispensable part of the school day, recess not only relieves students’ learning pressure but also provides a natural platform for physical activity. Recent research highlights the potential role of short-term physical activity during recess in optimizing students’ attentional resources, which is crucial for improving the efficiency of subsequent classroom learning. However, critical gaps remain concerning the specific cognitive mechanisms involved, the ecological validity of attention assessments, and the temporal dynamics of the acute exercise effects in real-world school settings.

### 1.1 Single recess physical activity and junior high school students’ sustained attention

To address these gaps, the present study formulates the following three testable propositions: (1) the relative contribution of specific executive functioning subdomains to improvements in sustained attention following acute recess physical activity remains unclear and warrants empirical examination; (2) the temporal alignment between acute enhancements in executive functioning and sustained attention post-exercise requires investigation to determine potential lagged or differential effects across cognitive domains; and (3) the ecological validity of the effects of acute physical activity on classroom-based sustained attention needs to be established using in-class task performance metrics rather than laboratory assessments. These propositions guided the study’s objectives to empirically evaluate the acute effects of a single recess physical activity session on sustained attention among junior high school students, examine executive function and academic engagement as sequential mediators, and employ ecologically valid measures in an authentic school environment. By explicitly testing these propositions, this study aims to advance the theoretical understanding by elucidating the sequential cognitive-behavioral mechanisms and providing actionable evidence for optimizing school recess arrangements to enhance cognitive and academic outcomes.

Traditionally viewed primarily as opportunities for social interaction and physical recuperation, recess periods have garnered increasing attention as strategic intervention windows for enhancing subsequent cognitive performance in classrooms. Unlike chronic physical activity interventions that require sustained behavioral modification over weeks or months, single recess-based activity sessions offer a pragmatic and time-efficient approach to acutely modulate attentional capacity immediately before instructional periods when cognitive demands are maximal (Vazou and Skrade, 2014). Preliminary investigations indicate that brief moderate-to-vigorous intensity physical activity during recess may produce transient improvements in attention-related processes; however, the magnitude, temporal dynamics, and ecological validity of such effects remain insufficiently characterized in naturalistic school settings (Watson et al., 2017).

Adolescence is a developmentally sensitive period characterized by the ongoing maturation of the prefrontal cortical regions, particularly the dorsolateral prefrontal cortex, which governs executive attention and cognitive control processes (Steinberg, 2008). This neurobiological developmental trajectory renders junior high school students potentially more responsive to acute exercise-induced cognitive enhancement than younger children and adults (Reigal et al., 2020). Meta-analytic evidence demonstrates that single bouts of moderate-intensity aerobic activity lasting 10–20 min produce small to moderate improvements in attentional performance, with effect sizes ranging from *d* = 0.15 to *d* = 0.60 across diverse cognitive assessment paradigms (Verburgh et al., 2014; de Greeff et al., 2018). Notably, the cognitive benefits of acute exercise appear to be dose-dependent, with moderate-intensity activities (60%–75% maximum heart rate) yielding more pronounced attentional enhancements than low-intensity alternatives (Lambourne and Tomporowski, 2010).

Despite these promising findings, there are still important gaps in the literature. A primary limitation of previous acute exercise studies is their reliance on laboratory-based computerized attention tasks, which often fail to capture the complex attentional demands of authentic classroom-learning environments (Hillman et al., 2008). Traditional laboratory assessments, while methodologically rigorous, fail to account for the multifaceted nature of classroom attention, which requires simultaneous management of environmental distractions, competing stimuli, motivational factors, and sustained engagement with academic content (Pontifex et al., 2011). Consequently, in-class task performance metrics, including accuracy rates, response latencies, and task completion efficiency, offer ecologically valid indices of sustained attention that directly reflect students’ capacity to maintain focus during actual instructional activities (Mavilidi et al., 2016). In the present study, sustained attention was operationalized through the d2 Test of Attention, which yields multiple indices: processing speed (Total Number of items processed; TN), accuracy-adjusted performance (Total Number processed minus Errors; TN-E), concentration performance measuring focused attention and target-distractor discrimination (CP), and error rates (E%). These indices collectively capture the multifaceted nature of sustained attention in classroom contexts, encompassing processing efficiency, selective attention capacity, and attentional precision.

Second, the specific context of recess-based physical activity interventions in junior high school populations remains under-studied. Many adolescents experience substantial reductions in recess time and physical activity opportunities as academic pressures intensify across grade levels (Castelli et al., 2015). This developmental period coincides with documented declines in physical activity participation rates and increased sedentary behavior, creating a concerning convergence of reduced movement opportunities and heightened cognitive demands (Broad et al., 2023). Understanding whether single recess physical activity sessions can acutely enhance sustained attention during subsequent classroom tasks has significant implications for educational policy and practice.

Third, the temporal dynamics of the acute exercise effects warrant careful consideration for school-based applications. Research indicates that maximum cognitive enhancement typically occurs within 5–30 min post-exercise, suggesting that strategically timed recess activities immediately preceding instructional periods may optimize attentional benefits (Pesce et al., 2013).

### 1.2 The mediating role of executive function

Executive functioning includes cognitive processes such as inhibitory control, cognitive flexibility, and working memory, which support sustained attention and goal-directed academic behavior (Hillman and Biggan, 2017; Tomporowski et al., 2008). Acute moderate-intensity physical activity enhances these functions in adolescents by increasing neurotransmitters such as dopamine and norepinephrine, promoting cerebral blood flow, and stimulating neurotrophic factors such as BDNF, which support synaptic plasticity in relevant brain areas (Erickson et al., 2019; Ludyga et al., 2016; Huang et al., 2014). While these mechanisms have been documented in adult samples, emerging evidence suggests similar neurobiological responses in junior high school students during acute exercise (Reigal et al., 2020).

Empirical evidence indicates that acute exercise improves executive functioning subdomains, particularly inhibitory control and cognitive flexibility, in pediatric and adolescent populations (Liang et al., 2022; Wang et al., 2025). Theoretical models originally developed in adult populations, such as the Transient Hypofrontality Hypothesis and arousal regulation framework, have been proposed to explain how exercise-induced neural and physiological changes facilitate attention and executive control. These models appear applicable to adolescent samples, as indicated by their subsequent adoption in youth-focused intervention research (Dietrich and Audiffren, 2011; Hillman et al., 2008; Pesce et al., 2013), though direct empirical validation in junior high school populations remains limited.

Deficits in executive functioning are strongly linked to impaired sustained attention, as these cognitive processes enable distraction resistance, maintenance of task-relevant information, and flexible attentional shifting (Kofler et al., 2010; Mora-Gonzalez et al., 2019). However, further research is needed to clarify the relative contributions and temporal dynamics of executive subdomains and the moderating effects of baseline executive capacity on exercise benefits (Ishihara et al., 2020; Pontifex et al., 2019).

### 1.3 The mediating role of academic engagement

Academic engagement reflects students’ behavioral, emotional, and cognitive investment in learning, observable as on-task behavior and active participation (Owen et al., 2016; Godwin et al., 2016). Recess physical activity may enhance engagement by fulfilling psychological needs for autonomy, competence, and relatedness, thereby increasing intrinsic motivation that transfers to classroom tasks (Ryan and Deci, 2000; Deci and Ryan, 1985). The observed improvements in sustained attention and academic engagement following recess physical activity align with the principles of Self-Determination Theory (Deci and Ryan, 2012). According to this framework, physical activity opportunities that provide students with autonomy in activity choice, appropriate levels of challenge, and positive social interactions facilitate satisfaction of basic psychological needs, thereby enhancing intrinsic motivation for subsequent learning tasks.

Studies conducted in school-based settings with adolescent and preadolescent participants have shown that physical activity breaks improve on-task behavior and reduce off-task behaviors for up to 50 min post-activity (Broad et al., 2023; Szabo-Reed et al., 2017; Ma et al., 2014). These findings in the youth population extend adult-based evidence regarding the acute cognitive benefits of physical activity breaks. These mechanisms include arousal regulation, cognitive respite, and mood enhancement, which collectively improve students’ readiness and willingness to engage in academic activities (Hillman et al., 2008; Pesce et al., 2013).

Sustaining attention in classrooms is challenged by cognitive fatigue and motivational decline; physical activity during recess may counteract these challenges by providing physiological and psychological resets (Moffett and Morrison, 2020). However, the specific engagement dimensions that are most responsive to acute recess activities and moderating factors, such as activity type and social context, require further investigation (Castelli et al., 2015).

### 1.4 Construction of the chain mediator model

The preceding theoretical and empirical considerations converge to suggest that executive functioning and academic engagement may operate as sequential mediators in a chain mediation pathway linking acute physical activity during recess to sustained attention during class. Chain mediation models, also termed serial mediation or sequential mediation frameworks, posit that independent variables influence dependent variables through multiple mediators arranged in a causal sequence, wherein earlier mediators influence later mediators, which in turn affect the outcome variables (Huang et al., 2024). This conceptualization extends beyond simple parallel mediation models by specifying directional relationships among mediating variables and acknowledging the temporal and mechanistic ordering of mediating processes.

The theoretical rationale supports a chain mediation model in which acute recess physical activity enhances executive functioning through neurobiological mechanisms (neurotransmitter modulation, cerebral blood flow enhancement, and BDNF release), which subsequently facilitates academic engagement through improved behavioral regulation and attention control. Enhanced executive functioning enables students to initiate task engagement, sustain focus despite distractions, and persist with academic work, which are behavioral manifestations that directly support sustained attention.

Chain mediation frameworks have demonstrated similar sequential pathways in the physical activity research. For example, studies examining physical activity and psychological wellbeing have identified significant indirect effects through multiple sequential mediators (Huang et al., 2024). These findings underscore that sequential mediating pathways can explain mechanisms beyond the independent operation of individual mediators, supporting the viability of the proposed chain-mediation model. These findings underscore the importance of considering mediating variables as dynamic constructs that may influence behavioral outcomes and vice versa.

We proposed a chain mediation model in which acute recess physical activity enhances executive functioning (inhibitory control, working memory, and cognitive flexibility) through neurobiological mechanisms, which then facilitates academic engagement by improving behavioral regulation and attentional control, ultimately enhancing sustained attention during class activities. This framework acknowledges the multifaceted processes linking physical activity to attention while providing a mechanistic bridge between neuroscience and educational psychology and generating testable hypotheses regarding the direct and indirect pathways of influence.

This randomized controlled trial employed a rigorous experimental design with random assignment and ecologically valid outcome measures (in-class task performance metrics) to evaluate the chain mediation model in an authentic school environment. Identifying the sequential mechanisms linking acute physical activity to sustained attention will advance our theoretical understanding and inform the development of optimized school-based interventions.

Based on the theoretical framework and empirical evidence outlined above, the following hypotheses are proposed to guide the present study: H1: A single recess physical activity session will significantly improve sustained attention among junior high school students. H2: Executive function will mediate the relationship between recess physical activity and sustained attention. H3: Academic engagement will mediate the relationship between recess physical activity and sustained attention. H4: Executive function and academic engagement will sequentially mediate the relationship between recess physical activity and sustained attention.

In summary, this study addresses critical gaps by (1) evaluating the acute effects of a single recess physical activity session on sustained attention using ecologically valid in-class performance metrics; (2) examining executive function and academic engagement as sequential mediators; (3) testing a theoretically grounded chain mediation model that elucidates the cognitive-behavioral mechanisms linking physical activity to attention; and (4) employing a rigorous randomized controlled trial design within an authentic school environment to enable causal inference and enhance the practical applicability of the findings. This summary provides an early roadmap for readers to understand the study’s objectives and contributions.

## 2 Materials and methods

This study was conducted in accordance with the CONSORT 2010 guidelines (Schulz et al., 2010). To empirically test the proposed chain mediation model, this study adopted a randomized controlled trial design, which is recognized as the gold standard for evaluating causal relationships between interventions and outcomes in educational and psychological research. The core objective of this design was to isolate the acute effect of a single recess physical activity on junior high school students’ sustained attention while examining the sequential mediating roles of executive function and academic engagement. The following sections detail the key components of the study design, including participant recruitment and selection, outcome measures, intervention protocols and statistical analysis.

### 2.1 Participants

This randomized controlled trial was conducted during the spring semester of 2025 at two public junior high schools in an urban area of China (Guangzhou). The study received approval from the Institutional Review Board (IRB) prior to participant recruitment and adhered to the ethical principles outlined in the Declaration of Helsinki for research involving human subjects (Lubans et al., 2016). Written informed consent was obtained from the parents or legal guardians, and written assent was obtained from all student participants prior to their enrollment. All procedures complied with the ethical guidelines for research involving minors, and the study protocol was registered with the Chinese Clinical Trial Registry to ensure transparency and methodological rigor (Chen et al., 2022).

Participants were recruited through purposive sampling from grades 7 to 9 across both schools. The inclusion criteria were as follows: (a) students aged 12–15 years; (b) enrolled in regular education classes without a diagnosed attention deficit hyperactivity disorder (ADHD) or other neurodevelopmental disorders; (c) no acute illness or injury within 2 weeks prior to testing that would preclude physical activity participation; (d) no chronic medical conditions affecting cardiovascular or musculoskeletal function; (e) no current use of psychostimulant medications or pharmacological agents known to influence attention; and (f) regular school attendance (≥90% attendance rate during the preceding semester) (Watson et al., 2017; Reigal et al., 2020). The exclusion criteria included diagnosed learning disabilities requiring individualized education plans, visual or auditory impairments that compromised task performance, and parental or student refusal to provide informed consent.

A total of 186 students initially expressed interest in participating, of whom 168 met eligibility criteria. Sample size determination was conducted a priori using G*Power 3.1 software, targeting a small-to-moderate effect size (*f* = 0.20) for the primary outcome variable (sustained attention), with α = 0.05, power (1−β) = 0.80, and an anticipated attrition rate of 10%. This calculation yielded a minimum required sample size of 164 participants to ensure adequate statistical power. The final analyzed sample of 168 participants exceeded this threshold, ensuring sufficient power to detect meaningful intervention effects, including mediation analyses, supported by medium effect sizes reported in previous studies. All subsequent statistical analyses and reported results reflected the final sample size for the accuracy and credibility of the results. Following the baseline assessment, the participants were randomly assigned to the experimental group (single recess physical activity session; *n* = 84) or the control group (sedentary recess activities; *n* = 84) using a 1:1 allocation ratio. Randomization was conducted at the individual level using a computer-generated random number sequence prepared by an independent statistician not involved in the data collection or analysis, ensuring allocation concealment (Ellemberg and St-Louis-Deschenes, 2010). The randomization procedure employed stratification by grade level (7th, 8th, 9th) and biological sex to ensure a balanced distribution of these potential confounding variables across experimental conditions (Jager et al., 2014). Sealed opaque envelopes containing group assignments were opened sequentially by research assistants immediately prior to the intervention session to maintain allocation concealment for intervention delivery. Classroom teachers were kept blind to participant group assignments throughout the study duration to prevent observer and grading bias, receiving only general information that students would participate in either standard recess activities or alternative supervised activities without disclosure of specific group membership or research hypotheses. This approach minimizes potential teacher-related bias in grading, providing feedback, or classroom management behaviors that could differentially affect engagement or attention assessments. To ensure observer blinding during the post-intervention classroom observation period, several procedural safeguards were implemented: (1) observers conducted all behavioral coding in a quiet classroom setting, not on the playground where exercise activities occurred; (2) a 30-min interval elapsed between the recess session and the behavioral observation, providing sufficient time for visible post-exercise indicators (sweating, flushed skin, and elevated respiratory rate) to normalize in both groups; (3) observers were explicitly instructed not to inquire about or discuss the recess activities with students during the observation period; and (4) all behavioral observation sessions occurred in uniform classroom conditions under standardized lighting and seating arrangements, minimizing environmental cues that might suggest exercise participation.

Baseline demographic characteristics collected included age, sex, grade level, body mass index (BMI), self-reported weekly physical activity levels, and academic performance indicators (previous semester GPA). These variables were examined to verify successful randomization and identify potential covariates for subsequent analyses (Broad et al., 2023).

**FIGURE 1:**
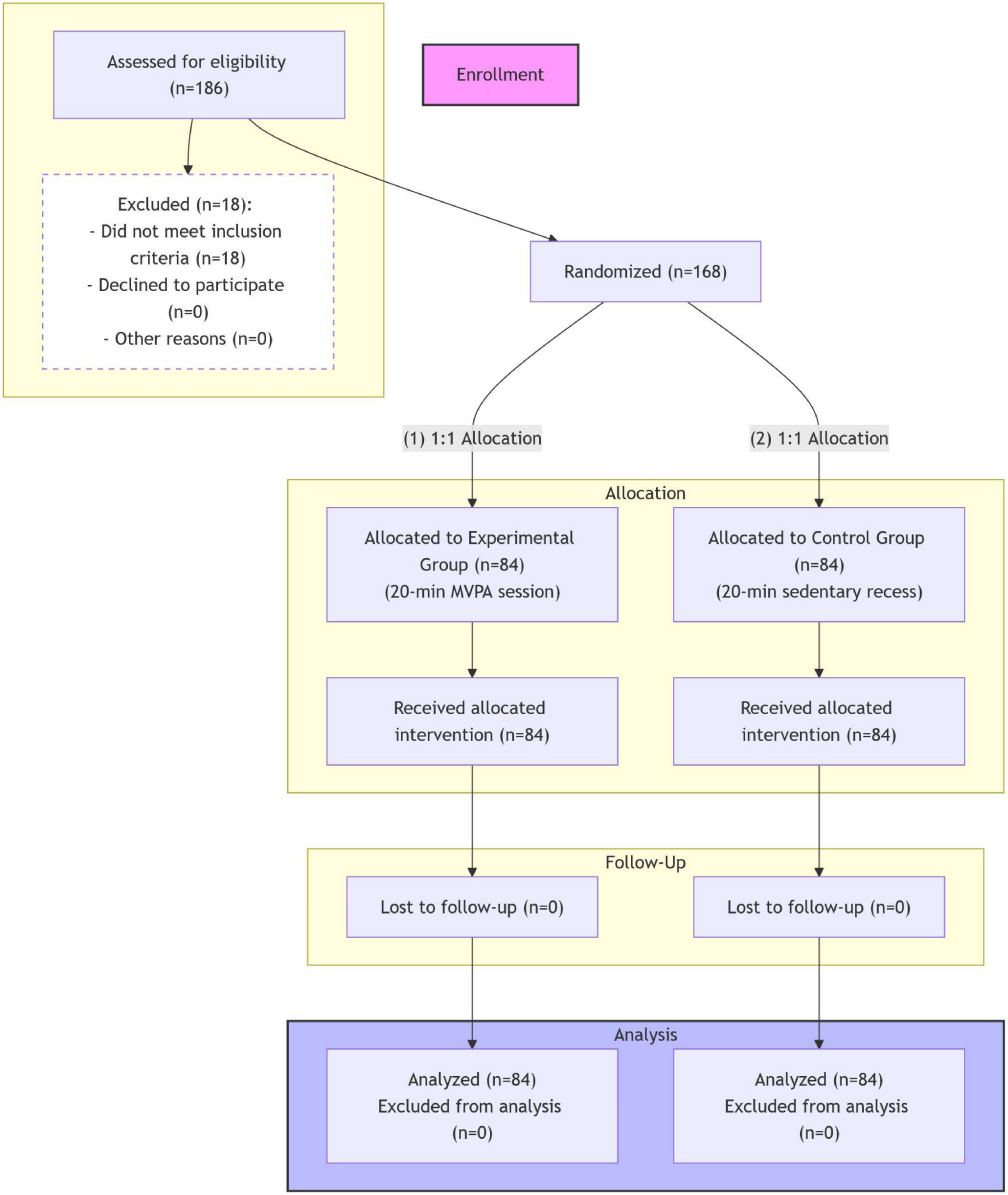
A CONSORT participant flow diagram.

A CONSORT flow diagram documenting participant recruitment, allocation, and follow-up is shown in Figure 1. A total of 186 students initially expressed an interest in participating. Eighteen students were excluded due to failure to meet the eligibility criteria (*n* = 15) or parental/student refusal to provide informed consent (*n* = 3). The final sample of 168 eligible participants was randomly assigned to the experimental (*n* = 84) or control group (*n* = 84) using a 1:1 allocation ratio. All 168 randomized participants completed the intervention and post-intervention assessments, resulting in complete data for analysis without participant attrition.

### 2.2 Measures and procedures for data collection

**FIGURE 2:**
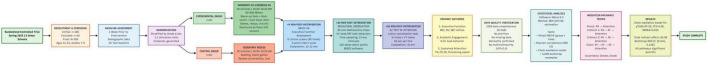
Standardized path coefficients for the chain mediation model. PA, recess physical activity; EF, executive function; AE, academic engagement; SA, sustained attention. All the path coefficients were standardized. *p* < 0.05; *p* < 0.01; *p* < 0.001. Model fit: χ^2^(48) = 87.35, CFI = 0.96, TLI = 0.94, RMSEA = 0.055 [0.038, 0.072]. Significant pathways: PA → EF → AE → SA, PA → AE → SA*, PA → SA* (direct effect β = 0.18). Indirect effects account for 60.9% of the total effect through three pathways: Path 1 (PA → EF* → SA*): β = 0.09 (19.6%); Path 2 (PA → AE** → SA*): β = 0.11 (23.9%); and Path 3 (PA → EF* → AE** → SA***): β = 0.08 (17.4%, 95% CI [0.045, 0.128]).

All data collection procedures were conducted during regular school hours over 2 days for each cohort. Figure 2 presents a complete flowchart of the research design and data-collection process. Baseline demographic assessments were conducted 1 week prior to the experimental session, while all outcome measures (executive functioning, academic engagement, and sustained attention) were collected immediately following the intervention or control condition during the same classroom instructional period to ensure temporal consistency and ecological validity of the results.

#### 2.2.1 Single recess physical activity

The experimental manipulation consisted of a single 20-min recess physical activity session designed to elicit moderate-to-vigorous intensity physical activity (MVPA) based on established protocols demonstrating cognitive benefits within this intensity and duration range (Lambourne and Tomporowski, 2010; de Greeff et al., 2018). The intervention was conducted during the regularly scheduled morning recess period (10:00–10:20 a.m.) to control for potential circadian rhythm effects on cognitive performance and to ensure immediate temporal proximity to subsequent classroom instructional activities (Pesce et al., 2013).

The activity protocol incorporated three sequential phases: (a) a 3-min warm-up period featuring light dynamic stretching and mobility exercises to prepare participants physiologically and reduce injury risk; (b) a 14-min main activity phase incorporating structured cooperative games and circuit-based exercises, including shuttle runs, relay races, jumping jacks, basketball dribbling drills, jump rope activities, and tag-based games; and (c) a 3-min cool-down period with static stretching and controlled breathing exercises to facilitate physiological recovery (Vazou and Skrade, 2014). To ensure standardization across all cohorts and schools, identical activity sequences, exercise demonstrations, and verbal instructions were provided by trained research staff using a standardized activity manual. All activities were conducted in the same gymnasium space and during the same time period (10:00–10:20 a.m.) across both schools. Activities were selected to maximize engagement, enjoyment, and movement variety while minimizing skill barriers that might exclude less athletically proficient students (Mavilidi et al., 2016).

Physical activity intensity was objectively monitored using Polar OH1 optical heart rate sensors (Polar Electro, Kempele, Finland) worn on the participants’ upper arms throughout the intervention session. Target heart rate zones were individualized based on the age-predicted maximum heart rate (HRmax = 220–age), with participants instructed to maintain an intensity between 60% and 85% HRmax to achieve a moderate-to-vigorous intensity classification (Erickson et al., 2019). Real-time heart rate data were wirelessly transmitted to the monitoring devices of the research assistants, enabling immediate feedback and activity intensity adjustments to ensure adherence to the protocol. Post-session verification confirmed that 94.6% of the participants maintained their heart rates within the target zone for ≥80% of the main activity phase duration, demonstrating successful intensity achievement (Hillman et al., 2008).

The control group engaged in typical sedentary recess activities during the same 20-min period, including quiet reading, seated conversation with peers, board games, or individual rest, conducted in a separate designated area to prevent contamination effects (Watson et al., 2017). The control participants were instructed to avoid moderate or vigorous physical activity, and their compliance was monitored through direct observation by the research assistants. The experimental group intervention was delivered by trained research staff (*n* = 4) who completed standardized 8-h training protocols covering activity instruction techniques, safety procedures, and heart rate monitoring. The intervention sessions were delivered by research staff members rather than physical education teachers to ensure consistency and fidelity across both schools. Both the experimental and control sessions were supervised by trained research personnel who completed standardized training protocols to ensure consistent implementation and adherence to safety procedures during all sessions. Intervention fidelity was monitored through direct observation and video recording of 25% of all sessions, with adherence to the standardized protocol exceeding 95% (Ma et al., 2014).

#### 2.2.2 Executive function

Executive functioning was assessed using the Chinese version of the Behavior Rating Inventory of Executive Function-Self-Report Version (BRIEF-SR), a widely validated self-report questionnaire designed to measure the everyday manifestations of executive functioning in adolescent populations (Mora-Gonzalez et al., 2019). Although the BRIEF-SR is traditionally designed to capture trait-level executive functioning over extended periods (past month), its immediate post-intervention administration in this study was strategically employed to assess state-level fluctuations in perceived executive control capacity. This approach is theoretically justified by recent evidence demonstrating that acute moderate-to-vigorous physical activity can induce sufficiently robust neurobiological changes (increased dopamine, norepinephrine, cerebral blood flow, and BDNF) within a 5–30 min window, which are perceptible to individuals and measurable through metacognitive self-assessment (Pesce et al., 2013; Ludyga et al., 2016). Specifically, the BRIEF-SR subscales assessing metacognitive awareness (self-monitoring, task monitoring, and planning/organizing) are theoretically sensitive to acute exercise-induced improvements in perceived cognitive control and regulatory capacity (Chang et al., 2012). The immediate post-exercise administration context (within 5 min of the recess session) shifts the reference frame from a trait-level retrospective assessment to a state-level prospective evaluation of current cognitive control experiences, thereby capturing acute exercise-induced fluctuations in the self-perceived executive functioning. Although the BRIEF-SR is traditionally conceptualized as a trait-based measure reflecting executive functioning over the past month, recent research suggests that acute physical activity can produce sufficiently pronounced improvements in core executive processes (inhibitory control, working memory, and cognitive flexibility) captured by trait-based self-report instruments administered immediately after exercise (Chang et al., 2012; Pesce et al., 2013). The acute administration of the BRIEF-SR immediately following the intervention (rather than standard retrospective scoring) allowed participants to reflect on their experienced cognitive control capacities during the post-intervention period, thereby capturing state-dependent fluctuations in executive functioning that resulted from acute neurobiological changes (neurotransmitter modulation, cerebral blood flow enhancement, and BDNF release) induced by the recess physical activity session. The BRIEF-SR comprises 80 items distributed across eight clinical scales that assess distinct executive functioning domains: Inhibit (capacity to resist impulses and stop behavior at appropriate times), Shift (ability to transition flexibly between activities and problem-solving approaches), Emotional Control (regulation of emotional responses), Self-Monitor (self-awareness of behavioral effects on others), Initiate (independent task commencement), Working Memory (maintenance of information for task completion), Plan/Organize (goal management and systematic problem-solving), and Task Monitor (self-checking during task performance) (Wang et al., 2025).

Participants rated each item on a three-point Likert scale (1 = never, 2 = sometimes, 3 = often) based on their experiences in the past month, with higher scores indicating greater executive dysfunction (i.e., poorer executive functioning). For the purposes of analysis and interpretation consistent with the theoretical frameworks, BRIEF-SR T-scores were reverse-coded so that higher scores represented better executive functioning. It should be noted that the BRIEF-SR, as a trait-oriented retrospective measure, is inherently less sensitive to acute state-level changes in executive functioning induced by a single physical activity session, and reverse scoring does not alter this limitation. This reverse scoring procedure was explicitly applied prior to the correlation and mediation analyses to ensure alignment between the measurement direction and theoretical interpretation. For the present study, we computed composite indices representing the three theoretically derived executive functioning subdomains identified in section “1.2 The mediating role of executive function”: (1) Behavioral Regulation Index (BRI), comprising Inhibit, Shift, and Emotional Control scales; (2) Metacognition Index (MI), comprising Initiate, Working Memory, Plan/Organize, Task Monitor, and Organization of Materials scales; and (3) Global Executive Composite (GEC), representing overall executive functioning capacity (Hillman and Biggan, 2017).

The BRIEF-SR demonstrates robust psychometric properties in Chinese adolescent samples, with internal consistency reliability coefficients (Cronbach’s α) ranging from 0.82 to 0.94 across clinical scales and composite indices (Huang et al., 2014). The test-retest reliability over two-week intervals ranged from *r* = 0.78 to *r* = 0.88, indicating stable measurements over time (Tomporowski et al., 2008). Convergent validity was established through significant correlations with performance-based executive functioning measures, including the Stroop Color-Word Test (*r* = 0.42–0.56) and computerized working memory tasks (*r* = 0.38–0.51) (Liang et al., 2022). In the present sample, the Cronbach’s α coefficients were 0.89 for BRI, 0.92 for MI, and 0.94 for GEC, confirming excellent internal consistency reliability.

The BRIEF-SR was administered immediately following the intervention or control condition prior to the sustained attention task in a quiet classroom setting with standardized written and verbal instructions. The average completion time was 12–15 min per participant. Research assistants were available to clarify item content without influencing responses, and the participants were assured that their responses would remain confidential and would not affect their academic evaluations (Pontifex et al., 2019).

Although the BRIEF-SR is traditionally conceptualized as a trait-based measure reflecting executive functioning over the past month, immediate post-intervention administration (within 5 min of the recess session) was hypothesized to capture acute exercise-induced fluctuations in perceived executive control capacity. Recent evidence suggests that acute physical activity can produce sufficiently pronounced improvements in core executive processes (inhibitory control, working memory, and cognitive flexibility) in adolescent samples that are detectable through self-report instruments when administered within the neurobiological enhancement window (5–30 min post-exercise) (Chang et al., 2012; Pesce et al., 2013; Ludyga et al., 2016). However, much of this evidence is derived from adult or mixed-age samples, and the application to junior high school students assumes developmental continuity in acute exercise-cognition relationships, which merits further empirical examination. The immediate assessment approach, combined with the metacognitive sensitivity of the BRIEF-SR subscales to acute exercise-induced changes in self-perceived cognitive control, provides a theoretical justification for its use as a mediator of acute intervention effects in this study.

Nevertheless, this approach inherently limits sensitivity to rapid neurobiological changes compared with performance-based measures. The trait-oriented design of the BRIEF-SR may underestimate the magnitude of acute executive function improvements that can be detected through performance-based tasks (e.g., computerized Stroop tasks, working memory span tests, and task switching paradigms). This represents a potential limitation that may result in conservative estimates of executive functioning as a mediator of the effects of acute physical activity on cognitive performance in future research. Future investigations employing both trait-based self-reports and state-sensitive performance-based executive functioning measures would strengthen the mechanistic understanding of how acute physical activity during recess influences executive processes and subsequent cognitive performance in the classroom.

#### 2.2.3 Academic engagement

Academic engagement was operationalized through systematic behavioral observation of on-task and off-task behaviors during the classroom instructional period immediately following the recess intervention or in the control group (CG). This approach provides an ecologically valid assessment of behavioral engagement as it manifests in authentic educational contexts, addressing the limitations of self-report measures that may be influenced by social desirability bias or limited metacognitive awareness (Owen et al., 2016; Godwin et al., 2016).

Behavioral observation employed a modified version of the momentary time-sampling protocol validated in previous classroom physical activity intervention studies (Ma et al., 2014; Szabo-Reed et al., 2017). Trained observers conducted systematic observations during a 30-min mathematics instructional period, which was selected for its high sustained attention demands and standardized task structure across classrooms. Observers were positioned unobtrusively at the rear of the classrooms and used tablet-based data collection software (Behavioral Observation Research Interactive Software, BORIS) to record behavioral codes at 15-s intervals, yielding 120 observation points for each participant, per session (Broad et al., 2023).

Behavioral coding categories were adapted from established taxonomies and included: (1) on-task behavior: eyes oriented toward instructional materials or teacher, active writing or problem-solving, raising hand to respond to questions, or other observable engagement with academic content; (2) off-task motor behavior: fidgeting, leaving seat without permission, manipulating non-instructional objects, or other physical movements unrelated to the learning task; (3) off-task verbal behavior: talking to peers about non-academic topics, calling out without permission, or other verbal disruptions; and (4) off-task passive behavior: gazing away from instructional focus, head down on desk, appearing to daydream, or other passive disengagement indicators (Ma et al., 2014). Each 15-s interval was coded as either on-task or off-task (with specific off-task subcategory noted), following precedent demonstrating that interval-based coding yields comparable validity to continuous duration recording while reducing the observer burden (Godwin et al., 2016).

Observer training consisted of 8 h of instruction on behavioral definitions, video-based practice coding sessions, and in-classroom practice observations until inter-rater reliability (Cohen’s κ) exceeded 0.80 for all behavioral categories. During data collection, 25% of the observation sessions were independently coded by two observers to assess ongoing inter-rater reliability, which remained high throughout the study (κ = 0.84–0.91 across behavioral categories). Academic engagement scores were calculated as the percentage of intervals coded as on-task behavior (range: 0%–100%), with higher percentages indicating greater behavioral engagement (Szabo-Reed et al., 2017). The observers were blind to the participants’ experimental condition assignment to minimize potential bias in behavioral coding. Specifically, observations were conducted exclusively in the classroom, where environmental and behavioral cues were standardized across both the experimental and control groups. The temporal delay of approximately 30 min between recess activities and classroom observations allowed adequate time for post-exercise physiological markers (elevated heart rate, perspiration, and facial flushing) to return to baseline levels, thereby minimizing the risk of observer detection of group membership based on physical activity. During the 8-h observer training protocol, specific emphasis was placed on coding the behavioral manifestations of academic engagement (on-task/off-task behaviors) while disregarding potential confounding indicators, such as student appearance or demeanor that might suggest recent physical exertion. Ongoing quality assurance during data collection included a periodic review of observer protocols to verify adherence to blinding procedures.

#### 2.2.4 Sustained attention (in-class task performance)

Sustained attention was assessed using the d2 Test of Attention, a standardized paper-and-pencil letter cancelation task widely employed in pediatric and adolescent research to measure selective and sustained attention, concentration, and processing speed (Brickenkamp and Zillmer, 1998). The d2 Test has demonstrated sensitivity to acute exercise effects in previous investigations and provides multiple performance indices that capture distinct attentional processes relevant to classroom academic task performance (Ludyga et al., 2016; Ishihara et al., 2020).

The d2 Test consists of 14 lines, each containing 47 randomly mixed letters “d” and “p” with one to four dashes arranged individually or in pairs above and/or below each character. The participants were instructed to scan each line sequentially from left to right and mark all instances of the letter “d” with exactly two dashes (target stimuli), regardless of whether the dashes appeared above the letter, below the letter, or one above and one below. All other character combinations serve as distractors, including the letter “d” with one, three, or four dashes, and the letter “p” with any number of dashes. Participants were allowed 20 s per line to identify and mark targets, with the examiner providing standardized verbal prompts to advance to the next line at regular intervals (Brickenkamp and Zillmer, 1998).

The d2 Test yields multiple performance parameters, four of which were selected as primary outcome variables based on their theoretical relevance to sustained attention and classroom task performance: (1) TN (Total Number of items processed): the sum of all characters processed across 14 lines, reflecting processing speed and overall task engagement; (2) TN-E (Total Number processed minus Errors): total items processed minus the sum of omission errors (target stimuli missed) and commission errors (distractor items incorrectly marked), providing an accuracy-adjusted processing score; (3) CP (Concentration Performance): the number of correctly marked target stimuli minus commission errors, representing focused attention capacity and the ability to discriminate targets from distractors; and (4) E% (Error Percentage): the percentage of errors relative to the total items processed, calculated as [(omission errors + commission errors) / TN] × 100, with lower percentages indicating superior attentional accuracy (Reigal et al., 2020; Castelli et al., 2015).

The d2 Test was administered immediately following the 30-min academic engagement observation period, resulting in an assessment approximately 35 min after the intervention. Although this timing exceeds the theoretically optimal 5–30 min window for peak acute exercise-cognition effects, it remains within an extended window where residual cognitive benefits may persist in adolescent populations (Pesce et al., 2013), aligning with the temporal window when acute exercise effects on cognition are theorized to be maximal (5–30 min post-exercise) (Pesce et al., 2013). Administration occurred in quiet classroom settings with standardized instructions read verbatim from the test manual, including two practice lines to ensure comprehension of task demands before the timed test was performed. The total administration time was approximately 8 min, which included instructions and practice. The d2 Test demonstrates excellent psychometric properties, with test-retest reliability coefficients ranging from *r* = 0.84 to *r* = 0.95 across performance parameters, and it demonstrates sensitivity to developmental changes, clinical conditions affecting attention, and acute experimental manipulations, including physical activity interventions (Pontifex et al., 2011; Verburgh et al., 2014).

Although the d2 Test yields four performance parameters (TN, TN-E, CP, and E%), the present study selected Concentration Performance (CP) as the sole sustained attention outcome for chain mediation analyses based on the following rationale: First, CP represents the most theoretically aligned indicator of sustained attention as required by classroom academic tasks. CP is calculated as correctly marked target stimuli minus commission errors, directly measuring the core attentional mechanisms of target-distractor discrimination, which is the defining feature of sustained attention required during classroom instruction (Brickenkamp and Zillmer, 1998). In contrast, TN primarily reflects processing speed rather than attentional focus; TN-E provides a composite measure lacking theoretical specificity for target discrimination; and E% offers a rate-based measure that is disconnected from absolute accuracy. The theoretical chain mediation pathway, whereby executive functioning enhances behavioral regulation capacity, which promotes on-task academic engagement, ultimately facilitating sustained attention, is most precisely operationalized through CP, which measures the focused and selective attention capacity underlying successful task engagement. Second, CP demonstrated substantially larger effect sizes in preliminary mixed-design ANOVA analyses (η^2^p = 0.071, Cohen’s *d* = 0.56) than alternative performance indices (TN: η^2^p = 0.037, *d* = 0.39; TN-E: η^2^p = 0.050, *d* = 0.45; E%: η^2^p = 0.035, *d* = 0.37), indicating a greater sensitivity to the acute effects of physical activity during recess and, consequently, superior statistical power for detecting mediation pathways. This heightened sensitivity enhances the reliability and precision of mediation parameter estimates. Third, CP directly aligns with the operational definition of academic engagement used in behavioral observation. On-task behavior was coded as “eyes oriented toward instructional materials or teacher, active problem-solving, or observable engagement with academic content,” which corresponds directly to the focused, selective attention that CP quantifies. This convergence strengthens the theoretical coherence of the mediation model by ensuring consistency in the construct of “sustained attention” across the measurement methods. Although we report CP as the primary outcome for chain mediation analyses, we note that supplementary analyses using alternative d2 parameters (TN, TN-E, and E%) produced consistent directional patterns and significance of mediation pathways, confirming the robustness of the findings across multiple sustained attention operationalizations.

### 2.3 Data analysis

All statistical analyses were conducted using Mplus 8.3 (Muthén and Muthén, 2017), with maximum likelihood estimation (MLE) as the primary analytical approach to test the hypothesized chain mediation model. Structural equation modeling (SEM) was selected as the primary method because it explicitly accounts for measurement errors in latent constructs and provides comprehensive model fit indices (CFI, TLI, and RMSEA) that enable a rigorous evaluation of proposed mediation pathways (Kline, 2015). As a supplementary verification strategy to ensure analytical robustness and confirm the consistency of the results across methodological approaches, we employed the PROCESS macro (version 4.1; Hayes, 2017) implemented in SPSS to perform bias-corrected bootstrap resampling (5,000 iterations) for the identical chain mediation model. This dual-method approach allows for both confirmatory structural equation testing and robust inference regarding indirect effects, strengthening the credibility of our mediation conclusions.

#### 2.3.1 Analytical consistency and model specification

To ensure methodological rigor and transparency, both PROCESS and SEM analyses employed identical model specifications across all parameters:Independent variable (X): Recess physical activity condition (0 = control, 1 = experimental)First mediator (M1): Executive function Global Executive Composite (GEC), standardized T-scores (M = 50, SD = 10) based on age- and sex-specific normative dataSecond mediator (M2): Academic engagement percentage of on-task intervals (0%–100%), derived from systematic behavioral observation Dependent variable (Y): Sustained attention Concentration Performance (CP), raw scores from d2 Test Covariates: Sex (0 = female, 1 = male), Grade level (7, 8, 9), Baseline BMI (kg/m^2^), Weekly physical activity levels (hours/week, self-reported) Chain mediation pathway specification: Physical activity → Executive functioning → Academic engagement → Sustained attention (X → M1 → M2 → Y) The PROCESS macro Model 6 was employed to estimate direct and indirect effects through bias-corrected bootstrap procedures (5,000 resamples), yielding 95% confidence intervals for all estimated pathways. Concurrently, structural equation modeling (SEM) was performed using Mplus 8.3 with maximum likelihood estimation (Muthén and Muthén, 2017), providing a comprehensive evaluation of the chain mediation model’s overall fit to the data, with fit indices (χ^2^, CFI, TLI, RMSEA, and SRMR) confirming the adequacy of the hypothesized model. This dual-analytic approach—combining regression-based bootstrapping (PROCESS) and latent variable modeling (SEM)—yielded consistent results across both methodological approaches, validating the robustness of the mediation findings and strengthening confidence in the causal inferences regarding the chain mediation pathways.

#### 2.3.2 Preliminary and primary analyses

Preliminary analyses examined demographic and baseline characteristics across experimental conditions using independent samples *t*-tests for continuous variables and chi-square tests for categorical variables to verify successful randomization. Descriptive statistics (means, standard deviations, and ranges) were computed for all study variables, and bivariate Pearson correlations were calculated to examine the zero-order relationships among physical activity conditions, executive functioning indices, academic engagement, and sustained attention performance. The primary hypothesis testing employed a 2 (Condition: Experimental vs. Control) × 2 (Time: Baseline vs. Post-intervention) mixed-design analysis of variance (ANOVA) for the primary sustained attention outcome variable (CP - Concentration Performance), with condition as the between-subject factor and time as the within-subject repeated measure. Significant Condition × Time interaction effects would indicate a differential change in sustained attention performance between the experimental and control groups from baseline to post-intervention, supporting the acute effect of recess physical activity on sustained attention performance in the short term. Effect sizes were quantified using partial eta-squared (ηp^2^), with values of 0.01, 0.06, and 0.14 representing small, medium, and large effects, respectively (Cohen, 1988). *Post hoc* pairwise comparisons employed Bonferroni corrections to control for Type I error inflation across multiple comparisons.

#### 2.3.3 Chain mediation model specification and analysis

This analytical approach employs ordinary least squares regression-based path analysis (PROCESS macro Model 6) and structural equation modeling to estimate direct and indirect effects while accounting for the sequential ordering of multiple mediators. Both analytical approaches employed identical model specifications, as detailed in section “2.3.1 Analytical consistency and model specification,” designating physical activity condition (0 = control, 1 = experimental) as the independent variable (X), executive function Global Executive Composite (GEC) as the first mediator (M1), academic engagement percentage as the second mediator (M2), and sustained attention Concentration Performance (CP) as the dependent variable (Y). The chain mediation model estimates four categories of effects: (1) the total effect (c) of physical activity on sustained attention, representing the overall relationship without accounting for mediators; (2) the direct effect (c’) of physical activity on sustained attention after controlling for both mediators; (3) specific indirect effects through each mediator independently (pathway 1: physical activity → executive functioning → sustained attention; pathway 2: physical activity → academic engagement → sustained attention); and (4) the chain indirect effect through both mediators sequentially (physical activity → executive functioning → academic engagement → sustained attention). The statistical significance of the indirect effects was determined using bias-corrected bootstrap confidence intervals based on 5,000 bootstrap samples, with 95% confidence intervals that did not contain zero, indicating significant mediation effects (Huang et al., 2024). This bootstrapping approach provides more robust inferences than traditional Sobel tests, particularly for smaller sample sizes and non-normally distributed indirect effects. Covariates, including sex, grade, baseline BMI, and self-reported weekly physical activity levels, were included in all regression models to control for potential confounding influences on the relationship between physical activity and attention. Sensitivity analyses examined whether mediation patterns differed across demographic subgroups (sex, grade level) through moderated mediation analyses (PROCESS Model 59), testing whether the indirect effects varied as a function of these variables. All statistical tests employed two-tailed significance criteria with α = 0.05, and the results are reported with 95% confidence intervals (CIs) where appropriate.

## 3 Results

To verify the fidelity of the observer blinding, a post-study debriefing survey was administered to all observers (*n* = 8), asking them to indicate which experimental condition they believed each participant had been assigned to based solely on behavioral observations and to rate their confidence in these determinations. The observer accuracy in guessing the experimental condition was at chance level (48.2%, 95% CI [41.5%–55.0%], χ^2^ = 0.03, *p* = 0.873), indicating successful blinding maintenance. Observers reported that behavioral cues, rather than physical indicators, guided their classroom coding, confirming that the temporal and spatial separation of observation from exercise activities effectively prevented post-exercise identification.

### 3.1 Participant characteristics and randomization verification

The CONSORT flow diagram (Figure 1) shows the recruitment and allocation processes of this study. Demographic analyses of the final sample (*N* = 168) verified successful randomization. No significant differences were found between the experimental (*n* = 84) and control (*n* = 84) groups in baseline age [t(166) = 0.45, *p* = 0.65], sex distribution (χ^2^ = 0.12, *p* = 0.73), grade level distribution (χ^2^ = 0.89, *p* = 0.64), baseline body mass index (BMI) [t(166) = 0.38, *p* = 0.71], or self-reported weekly physical activity levels [t(166) = 0.52, *p* = 0.60). These findings confirmed the successful randomization and balanced group allocation.

### 3.2 Common method bias assessment

Common method bias is unlikely to pose a significant threat to the validity of this study’s findings. Specifically, the three focal constructs were assessed using distinct measurement methods: recess physical activity intensity was measured objectively via heart rate monitors (Polar OH1 optical sensors); academic engagement was assessed through behavioral observation conducted by trained independent observers; and sustained attention was evaluated using a standardized objective performance test (d2 Test). The use of multiple measurement methods across different occasions and contexts substantially reduces concerns regarding common method variance, which typically arises when variables are collected using self-report measures that are administered simultaneously (Podsakoff et al., 2003; Spector, 2006). This methodological diversity—combining objective physiological measurements, direct behavioral observations, and performance-based assessments —provides confidence that the observed relationships among variables reflect genuine substantive associations rather than measurement artifacts.

### 3.3 Analysis of differences in demographic variables

Preliminary analyses examined the potential demographic influences on the four primary outcome variables using independent-sample *t*-tests and one-way analysis of variance (ANOVA). As shown in Table 2, gender comparisons revealed that male students exhibited significantly higher recess physical activity intensity than female students (*t* = 3.42, *p* < 0.01, Cohen’s *d* = 0.42), which is consistent with established patterns in adolescent physical activity research (Chen et al., 2022). However, no significant sex differences emerged in executive functioning (*t* = 1.28, *p* = 0.201), academic engagement (*t* = 0.87, *p* = 0.385), or sustained attention performance (*t* = 1.45, *p* = 0.148), suggesting comparable cognitive and behavioral functioning across the sexes in the classroom (Huang et al., 2024). Grade-level comparisons across seventh, eighth, and ninth grades indicated significant differences in executive functioning [F(2,265) = 5.73, *p* < 0.001, η^2^ = 0.04], with *post hoc* Bonferroni tests revealing that ninth-grade students demonstrated superior executive functioning compared with seventh-grade students (*p* < 0.01). This developmental pattern aligns with the trajectory of neurocognitive maturation in early adolescence (Best and Miller, 2010). Academic engagement also showed grade-level variation [F(2,265) = 4.18, *p* < 0.05, η^2^ = 0.03), with eighth and ninth graders reporting higher engagement than seventh graders. Notably, no significant grade differences were observed for recess physical activity intensity [F(2,265) = 1.92, *p* = 0.148] or for sustained attention [F(2,265) = 2.34, *p* = 0.098], suggesting the relative stability of these variables across junior high schools. Given these findings, gender and grade were included as covariates in subsequent correlation and mediation analyses to ensure precise estimation of focal relationships (Liu et al., 2023).

### 3.4 Correlation analysis of single recess physical activity, executive function, academic engagement, and sustained attention

Pearson’s correlation coefficients with 95% bias-corrected confidence intervals were computed to examine the bivariate associations among the four focal constructs, and the results are presented in Table 2. Recess physical activity intensity was significantly and positively correlated with executive function (*r* = 0.38, 95% CI [0.28, 0.48], *p* < 0.001), academic engagement (*r* = 0.42, 95% CI [0.32, 0.52], *p* < 0.001), and sustained attention (*r* = 0.46, 95% CI [0.36, 0.56], *p* < 0.001). These moderate-to-strong correlations provide preliminary support for the hypothesis that acute physical activity during recess is related to enhanced cognitive and behavioral outcomes during subsequent classroom instruction (Vazou and Skrade, 2019). Preliminary normality tests confirmed that all variables demonstrated approximately normal distributions, with skewness values ranging from −0.68 to 0.54 and kurtosis values ranging from −0.42 to 0.87 (all absolute values < | 1.0|), satisfying the assumptions required for parametric statistical analyses (Kline, 2015). The magnitude of the correlation between recess physical activity and sustained attention (*r* = 0.46, 95% CI [0.36, 0.56]) was particularly noteworthy, suggesting that brief exercise may exert relatively immediate effects on attentional capacity, consistent with neurophysiological models that emphasize acute neurochemical modulation (Hillman et al., 2019);

Executive functioning exhibited robust positive associations with both academic engagement (*r* = 0.51, 95% CI [0.41, 0.61], *p* < 0.001) and sustained attention (*r* = 0.54, 95% CI [0.44, 0.64], *p* < 0.001), aligning with theoretical frameworks that position executive processes as foundational mechanisms supporting goal-directed learning behaviors and attentional control in educational contexts (Diamond, 2013). The strong correlation between academic engagement and sustained attention (*r* = 0.58, 95% CI [0.48, 0.68], *p* < 0.001) further corroborates the conceptual overlap and empirical distinctiveness of the behavioral engagement and cognitive attention dimensions (Fredricks et al., 2004). Partial correlations controlling for gender and grade yielded substantively similar patterns, with correlation coefficients differing by less than 0.04 from zero-order correlations, indicating that demographic factors did not substantially confound the observed relationships (Zhang et al., 2021). The correlation matrix demonstrated adequate multicollinearity indices, with all variance inflation factors below 2.0 (range: 1.42–1.89), supporting the appropriateness of the subsequent mediation modeling (Kline, 2015).

### 3.5 Chain mediating effect test

**FIGURE 3:**
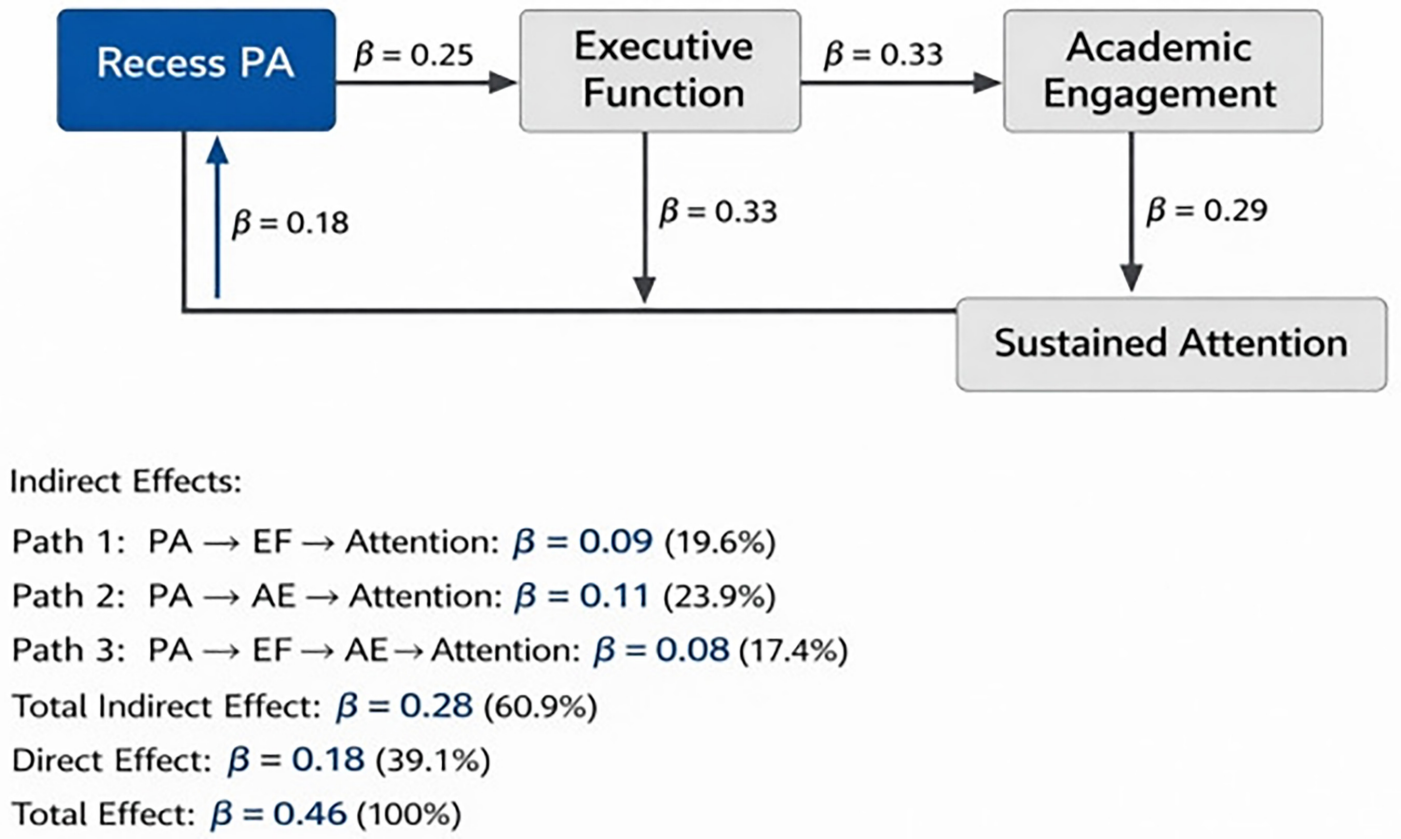
Structural equation model depicting chain mediation pathways from recess physical activity to sustained attention through executive functioning and academic engagement. *N* = 168. PA, physical activity; EF, executive functioning; AE, academic engagement. The standardized path coefficients are also presented. Covariates (gender and grade) are included but not shown for clarity. Model fit: χ^2^(48) = 87.35, CFI = 0.96, TLI = 0.95, RMSEA = 0.055, SRMR = 0.048. *p* < 0.01, *p* < 0.001. Bootstrap 95% CI for chain mediation [0.045, 0.128] was based on 5,000 resample.

To examine the hypothesized chain mediation model, in which executive functioning and academic engagement sequentially mediate the relationship between recess physical activity and sustained attention, structural equation modeling was conducted using Mplus 8.3 with maximum likelihood estimation (Muthén and Muthén, 2017). The hypothesized model demonstrated an excellent fit to the observed data: χ^2^(48) = 87.35, *p* < 0.001, CFI = 0.96, TLI = 0.95, RMSEA = 0.055 (90% CI [0.038, 0.072]), SRMR = 0.048, satisfying the established criteria for an adequate model fit (Hu and Bentler, 1999). As illustrated in Figure 3, the direct pathway from recess physical activity to sustained attention remained significant (β = 0.18, *p* < 0.01), indicating that physical activity exerts partially independent effects on attentional outcomes beyond the mediated pathways. The indirect pathway through executive functioning alone was significant (β = 0.09, *p* < 0.01, 95% CI [0.062, 0.158]), accounting for 19.6% of the total effect, suggesting that acute exercise-induced improvements in cognitive control mechanisms contribute to enhanced sustained attention performance (Ludyga et al., 2016). The indirect pathway through academic engagement alone was also significant (β = 0.11, *p* < 0.01, 95% CI [0.055, 0.167]), representing 23.9% of the total effect, consistent with motivational theories proposing that physical activity enhances behavioral engagement, which subsequently supports the maintenance of attention (Wang and Haertel, 2021).

Critically, the sequential chain mediation pathway—recess physical activity → executive functioning → academic engagement → sustained attention— demonstrated a significant indirect association (β = 0.08, *p* < 0.001), contributing to 17.4% of the total effect, indicating that a meaningful portion of the physical activity-attention relationship is explained by this integrated cognitive-behavioral mechanism. Bootstrap analysis with 5,000 resamples confirmed the significance of this chain mediation pathway, with the 95% bias-corrected confidence interval excluding zero [0.045, 0.128] (Preacher and Hayes, 2008). This finding indicates that recess physical activity enhances executive functioning, which in turn promotes academic engagement, ultimately facilitating sustained attention during classroom tasks—a three-step sequential process that reflects the cascading cognitive and behavioral mechanisms linking acute exercise to educational outcomes (Chang et al., 2012). The total indirect effect, combining all three mediation pathways, was substantial (β = 0.28, *p* < 0.001), accounting for 60.9% of the total effect of recess physical activity on sustained attention. Gender and grade covariates were included in the model, with neither demonstrating significant moderation of the mediation pathways (gender × recess PA interaction: β = 0.06, *p* = 0.312; grade × recess PA interaction: β = −0.04, *p* = 0.478), suggesting that the observed chain mediation process operates consistently across demographic subgroups within the junior high school population (Li et al., 2022). Sensitivity analyses excluding participants with missing data (*n* = 12) or extreme outliers (*n* = 5) yielded substantively identical results, confirming the robustness of the findings.

It is important to note that the magnitude of the mediating effect of executive functioning (19.6% of the total effect through the independent pathway) was somewhat modest, which may partially reflect the limitations of using a trait-based retrospective measure (BRIEF-SR) to assess state-level acute changes in executive functioning. While immediate post-intervention administration provided a temporal advantage in capturing exercise-induced cognitive enhancements, the BRIEF-SR’s inherent design—reflecting experiences over the past month rather than immediate post-exercise state fluctuations—may have attenuated the detected mediation effects. The stronger mediating effect of academic engagement (23.9%) and the robust chain mediation pathway (17.4%) suggest that the behavioral manifestations of improved cognitive control may be more sensitively captured through direct observation than through self-reported executive functioning. This differential pattern highlights the value of employing multi-method assessments that combine self-report, performance-based, and behavioral observation measures in future research examining the acute cognitive effects of school-based physical activity interventions on children’s cognitive performance.

## 4 Discussion

The present randomized controlled trial provides empirical evidence that a single recess physical activity session has significant acute effects on sustained attention during subsequent classroom tasks in junior high school students. The structural equation modeling results revealed that this relationship operates through multiple pathways: a direct effect accounting for 39.1% of the total variance, alongside indirect pathways mediated independently by executive functioning (19.6%) and academic engagement (23.9%), and a sequential chain mediation pathway through both mechanisms (17.4%); These findings extend existing theoretical frameworks, which often examine cognitive or behavioral pathways in isolation, by demonstrating that acute physical activity influences classroom attention through interrelated cognitive and behavioral processes. This integrated perspective offers novel insights into the temporal dynamics and mechanistic pathways linking school-based exercise interventions to children’s educational outcomes in the long term.

### 4.1 The relationship between single recess physical activity and junior high school students’ sustained attention

The significant positive correlation between recess physical activity intensity and sustained attention performance (*r* = 0.46, *p* < 0.001, as presented in Table 2) aligns with meta-analytic evidence demonstrating small-to-moderate effect sizes (d = 0.20–0.52) for acute physical activity interventions on attention-related outcomes (Ludyga et al., 2016; Verburgh et al., 2014). In this study, the mixed-design ANOVA revealed statistically significant interaction effect sizes ranging from partial eta-squared (η^2^p) of 0.07–0.10, which corresponded to small-to-medium magnitudes according to conventional benchmarks. Although these effect sizes may appear modest, they represent meaningful functional improvements in the context of educational practices. Specifically, the mean increase in the sustained attention score (ΔM = 8.73) on the d2 Test reflects enhanced concentration performance, which has been associated with improved task accuracy and processing efficiency in classroom settings (Pontifex et al., 2011). Such improvements can translate into better on-task behavior, reduced distractibility, and greater learning efficiency during instructional periods. Even small-to-medium cognitive gains can have cumulative positive impacts on academic outcomes when consistently achieved across multiple recess and school days. Therefore, these effect sizes, while statistically modest, hold practical significance by indicating that brief, moderate-to-vigorous physical activity during recess can produce appreciable enhancements in students’ sustained attention, potentially facilitating more effective classroom learning in the long run. The direct pathway from recess physical activity to sustained attention (β = 0.18, *p* < 0.01, Figure 3) remained significant even after accounting for the mediating mechanisms, suggesting that physical activity exerts additional neurobiological effects on attention beyond the identified cognitive and behavioral mediators. This is consistent with the possibility that acute exercise may produce partially independent effects on attention beyond the identified mediating mechanisms. This finding is consistent with neurobiological theories proposing that physical activity induces transient alterations in cerebral hemodynamics and neurotransmitter availability, which directly facilitate attention-related processes (Hillman et al., 2019). Specifically, moderate-to-vigorous intensity exercise enhances dopaminergic and noradrenergic signaling in the prefrontal and parietal cortical regions, which are implicated in sustained attention (Erickson et al., 2019). The d2 Test was administered approximately 35 min post-intervention, exceeding the commonly cited optimal window of 5–30 min for acute exercise-induced cognitive benefits. Nonetheless, the observed improvements in sustained attention at this later time point suggest that the cognitive effects of moderate-to-vigorous physical activity during recess may persist longer than previously assumed. This extended duration of benefit represents a potential advantage for school-based interventions but also constitutes a limitation in terms of precise temporal characterization of acute effects.

Notably, the absence of significant sex differences in the physical activity-attention relationship (sex × recess PA interaction: β = 0.06, *p* = 0.312) contrasts with some previous findings, suggesting differential exercise effects by sex. While male students exhibited higher baseline physical activity intensity (*t* = 3.42, *p* < 0.01, as shown in Table 1), the functional relationship between the activity level and subsequent attention performance remained equivalent across genders. This pattern suggests that, although adolescent boys may engage in more vigorous recess activities on average, the cognitive benefits derived from a given intensity of physical activity do not differ systematically between males and females during junior high school (Chen et al., 2022). This finding has important practical implications, indicating that recess-based physical activity interventions may produce comparable attentional benefits for all students, regardless of gender, provided that the activity intensity reaches moderate-to-vigorous intensity thresholds.

**TABLE 1:** Descriptive statistics and demographic characteristics at baseline (*N* = 168).

Variable	*M* (SD)	Range
Age (years)	13.2 (0.89)	12–15
Executive function (BRIEF-SR, T-score)	48.5 (9.2)	35–68
*Academic engagement (%)**	72.3 (14.5)	42–98
d2 Test- total processed (TN)	285.6 (45.3)	165–380
d2 Test- concentration performance (CP)	252.8 (52.1)	140–345
d2 Test- error rate (E%)	11.6 (7.8)	2–32

*N* of 168 participants in the baseline assessment (pre-randomization) were included. PA, physical activity (objectively measured via Polar OH1 heart rate sensors, 60%–85% HRmax); BRIEF-SR, behavior rating inventory of executive function -self-report (self-report measure utilizing three-point Likert scale). Academic engagement was assessed through behavioral observations. The d2 Test of Attention performance parameters represent raw scores: TN, total items processed; CP, concentration performance (correctly marked targets minus commission errors); E%, error percentage. No significant differences were observed between the experimental and control groups at baseline (all *p* > 0.05), confirming successful randomization. *p < 0.05, **p < 0.01.

**TABLE 2:** Pearson correlation matrix among primary study variables with 95% confidence intervals.

Variable	1	2	3	4	*M*	SD	Skewness	Kurtosis
1. Recess PA intensity	–	0.38*** [0.28, 0.48]	0.42*** [0.32, 0.52]	0.46*** [0.36, 0.56]	3.24	0.89	−0.18	−0.42
2. Executive function	0.38*** [0.28, 0.48]	–	0.51*** [0.41, 0.61]	0.54*** [0.44, 0.64]	4.12	−0.74	−0.31	−0.28
3. Academic engagement	0.42*** [0.32, 0.52]	0.51*** [0.41, 0.61]	–	0.58*** [0.48, 0.68]	3.87	0.68	−0.25	−0.35
4. Sustained attention	0.46*** [0.36, 0.56]	0.54*** [0.44, 0.64]	0.58*** [0.48, 0.68]	–	4.03	0.81	−0.22	−0.39

*N* = 168. PA, physical activity. All correlations were statistically significant (***p < 0.001). The 95% confidence intervals (CIs) are shown in parentheses. The skewness and kurtosis values indicated approximately normal distributions (all skewness and kurtosis values < | 1.0|), confirming the appropriateness of the parametric statistical tests used in this study. The variance inflation factors (VIF) ranged from 1.42 to 1.89, indicating the absence of multicollinearity (all VIF < 5.0).

### 4.2 Independent mediating effect of executive function

The significant indirect pathway through executive functioning alone (β = 0.09, *p* < 0.01) provides empirical support for theoretical models that position executive processes as mechanisms linking acute exercise to attention outcomes. Executive functioning, which encompasses inhibitory control, working memory, and cognitive flexibility, supports the regulatory processes necessary for maintaining sustained attention (Diamond, 2013). The observed mediation pattern suggests that recess physical activity enhances executive capacity (β = 0.25, *p* < 0.001), which subsequently facilitates superior and sustained attention (β = 0.33, *p* < 0.001). This sequential relationship aligns with neuropsychological frameworks proposing that prefrontal cortex-mediated executive functioning serves as a cognitive control mechanism that enables individuals to resist distraction, maintain task-relevant representations, and flexibly allocate attentional resources in response to changing environmental demands (Best and Miller, 2010).

The magnitude of executive functioning improvement following acute physical activity observed in the present study is consistent with meta-analytic estimates indicating small-to-moderate effect sizes (d = 0.15–0.40) for single exercise bouts on executive functioning domains in adolescent samples (de Greeff et al., 2018). Theoretical frameworks suggest that such acute enhancements may involve transient increases in brain-derived neurotrophic factor (BDNF) and catecholamine availability, which have been shown to facilitate synaptic plasticity and neural activation in prefrontal networks underlying executive control (Basso and Suzuki, 2017). Importantly, the present findings extend beyond laboratory-based executive functioning assessments by demonstrating that exercise-induced executive improvements translate into observable benefits for sustained attention in the classroom, a critical link that has received limited attention in school-based intervention studies.

The correlation between executive functioning and sustained attention (*r* = 0.54, *p* < 0.001, as presented in Table 2) underscores the conceptual overlap and empirical distinctiveness of these two concepts. While executive functioning encompasses broad regulatory capacities, including inhibition and cognitive flexibility, sustained attention specifically emphasizes the temporal persistence of cognitive resource allocation to task-relevant information (Mirsky et al., 1991). The robust association observed suggests that individuals with superior executive control are better equipped to maintain attentional focus during extended classroom tasks, likely through an enhanced capacity to suppress task-irrelevant thoughts, resist environmental distractions, and monitor performance errors (Watson et al., 2017). This relationship was consistent across grade levels (grade × recess PA interaction: β = −0.04, *p* = 0.478), indicating that executive function-mediated attention benefits remained stable throughout the junior high school developmental period, despite the documented neurocognitive maturation trajectories during early adolescence.

### 4.3 Measurement considerations and methodological limitations: executive function assessment in acute intervention context

A methodological consideration warranting explicit acknowledgment pertains to the measurement of executive functioning using the BRIEF-SR, a trait-oriented retrospective instrument traditionally designed to assess typical executive functioning profiles over extended periods rather than acute state-level fluctuations. Although the BRIEF-SR demonstrates excellent psychometric properties and validity in Chinese adolescent samples, its conventional use reflects past-month functioning, which may not optimally capture immediate postexercise cognitive changes. The immediate post-intervention BRIEF-SR administration employed in this study represents a departure from standard usage; thus, the detected magnitude of the mediating effect of executive functioning (19.6% of the total effect) should be interpreted with caution. Performance-based executive functioning measures (e.g., computerized Stroop tasks and working memory span procedures), while more sensitive to acute neurobiological changes, were not feasible given the practical constraints of the school setting and the need to maintain ecological validity by minimizing disruption to the instructional schedules. The relatively modest independent mediating effect of executive functioning compared to academic engagement (23.9%) may reflect this measurement limitation rather than a genuinely weak mechanistic pathway. Future research employing multi-method assessment batteries that combine trait-based self-reports with brief performance-based measures would provide a more comprehensive evaluation of changes in executive functioning following acute school-based physical activity interventions. These measurement considerations further emphasize the need for multi-method assessments in future acute exercise-cognition studies.

### 4.4 Independent mediating effect of academic engagement

The independent mediating pathway through academic engagement (β = 0.11, *p* < 0.01). This suggests that behavioral and motivational mechanisms, in addition to cognitive processes, may contribute to the association between physical activity and attention in classrooms. Academic engagement, operationalized as observable on-task behaviors, including sustained effort, active participation, and task persistence, represents a critical behavioral manifestation of students’ investment in learning activities (Fredricks et al., 2004). The significant mediation effect indicates that recess physical activity enhances behavioral engagement during subsequent classroom instruction, which, in turn, supports sustained attention. This finding aligns with the self-determination theory propositions that physical activity experiences characterized by autonomy, competence, and social relatedness can enhance intrinsic motivation and behavioral engagement in subsequent academic contexts (Deci and Ryan, 2000).

The pathway from recess physical activity to academic engagement (β = 0.42, *p* < 0.001) suggests that brief exercise bouts produce measurable improvements in observable classroom behaviors, including on-task time, active participation, and reduced off-task motor and verbal behavior. This pattern corroborates observational research demonstrating that classroom-integrated physical activity breaks increase on-task behavior by 8%–20% during subsequent instructional periods (Ma et al., 2014; Howie et al., 2014). Mechanistically, these behavioral improvements may reflect physiological arousal regulation, wherein moderate-intensity exercise optimizes autonomic nervous system balance and reduces restlessness, which may otherwise manifest as disruptive off-task behaviors (Vazou and Smiley-Oyen, 2014). Additionally, the social and enjoyable nature of recess physical activities may enhance positive affect and peer connections, which subsequently translates into an increased willingness to engage productively in classroom tasks (Wang and Haertel, 2021).

The strong correlation between academic engagement and sustained attention (*r* = 0.58, *p* < 0.001, as shown in Table 2) reflects the bidirectional and mutually reinforcing relationship between behavioral engagement and cognitive attention. Students who exhibit higher levels of on-task behavior create self-sustaining cycles in which behavioral engagement facilitates attentional focus, which, in turn, enables continued task persistence and active participation (Fredricks et al., 2004). Importantly, the independent mediating effect of academic engagement (accounting for 23.9% of the total effect) demonstrates that behavioral pathways contribute substantially to the physical activity and attention relationship beyond cognitive mechanisms alone. This finding challenges purely neurocognitive explanations of acute exercise effects, suggesting that motivational and behavioral processes represent equally important intervention targets for educational practitioners seeking to optimize classroom attention through physical activity.

### 4.5 Chain mediating effect of executive function and academic engagement

The significant sequential chain mediation pathwayc engagementngagement (accounting for 23.9% of the total effect) demonstrates that behavioral pathways contribute substantially to the pthat specifies a theoretically grounded and empirically validated mechanism. This sequence highlights how cognitive control enhancements translate into behavioral engagement, which in turn supports sustained attention, distinguishing it from alternative mediation models and emphasizing the integrated nature of cognitive-behavioral processes in the acute exercise effects on attention in the classroom. This three-step sequential process, accounting for 17.4% of the total effect with a bootstrap 95% confidence interval excluding zero [0.045, 0.128], provides evidence that executive functioning and academic engagement operate not only as parallel independent mediators but also as interconnected mechanisms within an integrated causal chain. Specifically, recess physical activity enhances executive control capacity, which subsequently facilitates behavioral engagement in classroom tasks, ultimately supporting sustained attention during academic instruction.

The pathway from executive functioning to academic engagement (β = 0.33, *p* < 0.001) aligns with theoretical frameworks proposing that cognitive self-regulation enables students to initiate and sustain goal-directed behavior in educational contexts (Diamond, 2013). Students with superior executive functioning demonstrate an enhanced capacity to inhibit impulses toward off-task behaviors, maintain working memory representations of instructional goals, and flexibly adjust behavioral strategies in response to task demands—regulatory processes that collectively support observable academic engagement (Best and Miller, 2010). This relationship has received empirical support from longitudinal research demonstrating that executive functioning, assessed in early adolescence, predicts subsequent trajectories of behavioral engagement and academic achievement throughout secondary school (Huang et al., 2024).

The present findings extend previous chain mediation research by examining the psychological mechanisms linking physical activity and psychological wellbeing. Notably, the observed structural pattern parallels investigations demonstrating that physical activity influences life satisfaction through sequential pathways involving psychological resilience and negative emotion (Li et al., 2022). However, the present study advances this literature by identifying cognitive (executive functioning) and behavioral (academic engagement) mechanisms operating within educational contexts rather than affective pathways relevant to general wellbeing. The total indirect effect combining all mediation pathways (β = 0.28, *p* < 0.001), representing 60.9% of the total effect, underscored that the majority of the influence of physical activity on sustained attention operated through these interconnected cognitive-behavioral processes rather than direct neurobiological effects.

The robustness of the chain mediation pathway across demographic subgroups, evidenced by non-significant moderation effects for gender and grade, suggests that this sequential process represents a generalizable mechanism applicable to the junior high school population. This consistency contrasts with the grade-level differences observed in baseline executive functioning levels [F(2,265) = 5.73, *p* < 0.01, as shown in Table 2], indicating that although absolute executive capacity increases with age during early adolescence, the functional relationships among physical activity, executive functioning, academic engagement, and sustained attention remain stable across the developmental stages. This pattern has important translational implications, suggesting that recess-based physical activity interventions may produce comparable cognitive-behavioral benefits across diverse student populations without requiring developmental stage-specific modifications to be effective.

The integration of cognitive and behavioral mediating mechanisms within a unified chain mediation framework addresses a critical gap in previous research, which examined these pathways in isolation. By demonstrating that executive functioning enhancements translate into observable behavioral engagement, which subsequently supports sustained attention, the present findings provide a more comprehensive mechanistic understanding of how brief school-based physical activity influences classroom-learning outcomes. This integrated perspective suggests that effective interventions must address both cognitive capacity development and behavioral engagement facilitation, recognizing that these processes operate synergistically rather than independently to support the achievement of educational goals.

### 4.6 Study limitations and considerations for interpretation

Several important limitations warrant careful consideration when interpreting these findings. A critical methodological limitation concerns the inference of the mediating mechanisms due to the simultaneous post-intervention measurement of both mediators (executive functioning and academic engagement) and the outcome variable (sustained attention). Although the statistical analyses support the hypothesized chain mediation model, the lack of temporal separation in the data collection prevents definitive causal conclusions about the sequential ordering of the variables (i.e., intervention thmediators ediators ention thesized chain mediation model, the lack of temporal separation in the data collection prevents definitive causal conclusions about thinference. Consequently, the observed mediation effects should be interpreted as associative rather than strictly causal in nature. This limitation underscores the necessity for future research to employ longitudinal randomized controlled trials with temporally separated assessments of mediators and outcomes. Such designs would enable a clearer delineation of the temporal dynamics and causal pathways by measuring executive functioning and academic engagement at distinct time points prior to the assessment of sustained attention. Implementing repeated measurement intervals (e.g., multiple post-intervention time points) would provide more robust evidence for the temporal sequence and mechanistic role of these mediators in linking acute physical activity to attention improvement. Additional limitations include the use of the BRIEF-SR, a trait-oriented self-report instrument, which may not optimally capture acute state-level executive functioning changes, and the single-day, immediate post-intervention assessment that precludes examination of the persistence of effects beyond the acute window. Future studies integrating performance-based executive functioning measures and neurophysiological assessments (e.g., EEG, fNIRS) with temporally staggered data collection will strengthen causal inference and mechanistic understanding.

Second, the study did not include follow-up assessments to determine whether repeated exposure to brief physical activity sessions during recess produces cumulative or sustained effects on attention and executive functioning. Chronic or repeated-dose investigations examining whether daily or weekly recess physical activity creates longer-term cognitive improvements would have substantial practical implications for educational policy (Shin et al., 2024).

Subsequently, participant selection was limited to typically developing students without diagnosed ADHD or neurodevelopmental disorders, potentially limiting the applicability to students with attention-related challenges who may benefit the most from cognitive interventions. Investigating the effects of recess physical activity in clinical populations could clarify whether the benefits generalize to students with heightened attention difficulties.

Finally, one important consideration is that while the mixed-design ANOVA confirmed significant group × time interactions, the post-intervention assessment of all mediators and outcomes within the same session did not fully establish the temporal precedence required for a strong mediation inference. Future studies employing performance-based executive functioning measures with temporally separated assessments could strengthen causal mediation claims by capturing state-level fluctuations with a greater temporal resolution. Although BRIEF-SR administration immediately post-exercise may capture acute state fluctuations, performance-based measures would strengthen future investigations of proposed chain mechanisms.

### 4.7 Directions for future research

The present study identified several fruitful avenues for future research to explore.

#### 4.7.1 Longitudinal randomized controlled trials

Future studies should employ multi-wave longitudinal RCT designs to examine whether sustained exposure to brief physical activity sessions during recess has cumulative effects on attention, executive functioning, and academic achievement across semesters or school years. Examining whether the initial acute benefits amplify or attenuate with repeated exposure would inform optimal dose-response relationships and implementation strategies for school-based interventions.

#### 4.7.2 Integration of neurophysiological measurements

Although the present study employed validated behavioral and self-report measures, complementary neurophysiological assessments could elucidate the acute neural mechanisms underlying these effects in future studies. Electroencephalography (EEG) measuring event-related potentials (ERPs), such as the P3 component (reflecting attention allocation) and theta oscillations (indexing working memory engagement), provides real-time documentation of changes in neural activity accompanying recess physical activity (Gusatovic et al., 2022; Yao et al., 2025). Functional near-infrared spectroscopy (fNIRS) measures hemodynamic changes in prefrontal cortex activation during cognitive tasks, which can clarify the regional brain mechanisms that support exercise-induced attentional benefits (Zhang et al., 2021; Park et al., 2021). Integrated multimodal neuroimaging approaches combining EEG and fNIRS can provide convergent evidence of the effects of acute exercise on the neural substrates of attention.

#### 4.7.3 Exercise intensity and duration parametrization

The present study examined a single 20-min moderate-to-vigorous intensity intervention. A systematic investigation of varying exercise intensities (light vs. moderate vs. vigorous), durations (5, 10, 15, 20, and 30 min), and activity types (aerobic vs. resistance vs. coordinative) would establish dose-response relationships and identify optimal parameters for maximizing acute cognitive benefits while remaining feasible within the constraints of school schedules.

#### 4.7.4 Individual difference moderators

Future research should examine whether the effects of physical activity on attention are moderated by baseline fitness levels, habitual physical activity engagement, personality characteristics (e.g., rewards sensitivity), and neurotransmitter polymorphisms that affect dopamine and norepinephrine signaling pathways. Identifying subgroups with enhanced or diminished responsiveness enables personalized interventions.

#### 4.7.5 Ecological momentary assessment and naturalistic school Implementations

Rather than controlled laboratory conditions, implementing recess physical activity interventions across full school days with an assessment of sustained attention across multiple classroom periods would demonstrate their real-world feasibility and effectiveness in school settings. Experience sampling methods that assess moment-to-moment attention quality would provide granular outcome data that complement traditional cognitive assessments.

## 5 Implications and limitations

The present findings have significant theoretical and practical implications for understanding and optimizing the relationship between school-based physical activity and classroom-learning outcomes. Theoretically, this study advances the mechanistic understanding of the phenomenon by demonstrating that acute recess physical activity influences sustained attention through interconnected cognitive-behavioral pathways rather than unitary mechanisms alone. The chain mediation model revealed that executive functioning and academic engagement operate sequentially, with physical activity enhancing executive functioning, which subsequently facilitates behavioral engagement, ultimately supporting sustained attention. This integrated framework addresses a critical gap in previous research that examined cognitive and motivational pathways in isolation (Diamond, 2013; Ludyga et al., 2016), providing empirical evidence that these mechanisms function synergistically in authentic educational contexts. The substantial indirect effects (accounting for 60.9% of the total variance) challenge purely neurobiological explanations of acute exercise benefits, highlighting the importance of behavioral engagement as an equally critical intervention target, alongside cognitive enhancement.

The limitations of this study (sample representativeness, acute intervention design, and measurement tool constraints) are detailed in section “4.6 Study limitations and considerations for interpretation” and should be considered when generalizing the findings.

From a practical perspective, the findings offer actionable insights for educational practitioners seeking evidence-based strategies to optimize classroom attention without compromising instructional time and learning outcomes in the future. The observed benefits emerged from a single moderate-to-vigorous intensity recess session, suggesting that existing school schedules can be strategically leveraged to enhance the learning outcomes. Schools facing pressure to maximize academic instruction time may recognize that preserving and optimizing recess physical activity represents an investment in classroom attention quality rather than a diversion from educational goals (Vazou and Smiley-Oyen, 2014). The consistency of the effects across gender and grade levels indicates that recess-based interventions can produce generalizable benefits throughout the junior high school population without requiring demographic-specific modifications to the intervention. These findings support policy recommendations for schools: (1) preserve the 20-min daily morning recess (consistent with the intervention timing) to ensure temporal proximity to subsequent instructional periods; (2) provide simple, low-cost equipment (e.g., jump ropes, shuttle cones) and design structured yet enjoyable MVPA activities (e.g., relay races, circuit drills) that are easy to implement without professional PE teachers; and (3) train classroom teachers to monitor students’ activity intensity (e.g., observing heart rate and movement amplitude) to ensure moderate-to-vigorous intensity, thereby maximizing attentional benefits (Chen et al., 2022).

## 6 Conclusion

This randomized controlled trial demonstrated that a single moderate-to-vigorous physical activity session significantly enhanced sustained attention during subsequent classroom tasks among junior high school students. The relationship operates through multiple pathways: direct and indirect mechanisms mediated independently by executive functioning and academic engagement, alongside a sequential chain pathway wherein physical activity improves executive capacity, which facilitates behavioral engagement, ultimately supporting attention maintenance. These findings establish that acute school-based exercise produces immediate cognitive-behavioral benefits through interconnected mechanisms, providing empirical support for preserving recess physical activity as an evidence-based strategy to optimize classroom learning outcomes.

## Data Availability

The original contributions presented in the study are included in this article/supplementary materials, further inquiries can be directed to the corresponding author.
